# Modelling of a genetically diverse evolution of Systemic Mastocytosis with Chronic Myelomonocytic Leukemia (SM-CMML) by Next Generation Sequencing

**DOI:** 10.1186/2162-3619-3-18

**Published:** 2014-07-11

**Authors:** Markus Rechsteiner, Rouven Müller, Tanja Reineke, Jeroen Goede, Annette Bohnert, Qing Zhong, Markus G Manz, Holger Moch, Peter J Wild, Dieter R Zimmermann, Marianne Tinguely

**Affiliations:** 1 Institute of Surgical Pathology, University Hospital Zurich, Zurich, Switzerland; 2 Clinics for Hematology, University Hospital Zurich, Zurich, Switzerland; 3 Kempf and Pfaltz, Histological Diagnostics, Seminarstr. 1, 8042 Zurich, Switzerland

**Keywords:** Chronic myelomonocytic leukemia, Systemic mastocytosis, SM-CMML, Next Generation Sequencing, *c-KIT* mutation, *TET-2* mutation, *RUNX1* mutation

## Abstract

**Background:**

Systemic mastocytosis (SM) is a heterogenous, clonal mast cell (MC) proliferation, rarely associated with clonal hematologic non-mast cell lineage disease (SM-AHNMD). *KIT*^D816V^ is regarded as driver-mutation in SM-AHNMD.

**Methods:**

DNA isolated from peripheral blood (PB) of an SM-CMML patient was investigated with targeted next generation sequencing. Variants were verified by Sanger sequencing and further characterized in the SM part of the bone marrow trephine (BMT), normal tissue, and FACS sorted PB cell subpopulations.

**Findings:**

Low coverage deep-sequencing (mean 10x) on a GS 454 Junior revealed two as yet unreported SNVs (*CBFA2T3* and *CLTCL1*), both germ-line mutations. High coverage (mean 1674x) targeted re-sequencing on an Ion Proton revealed 177 variants in coding regions. Excluding SNPs, the final list comprised 11 variants. Among these, *TET2* (p.Thr1027fs, p.Cys1263Ser) and *RUNX1* (p.Asn109Ser) were identified in in the peripheral blood and the SM part of BMT, but not in normal tissue. Furthermore, Sanger sequencing of PB cells revealed similar signal intensities for both *TET2* mutations in FACS sorted CD34+ precursor cells and CD16+ granulocytes comparable to signals in the SM part of BMT. In contrast, *RUNX1* exhibited a double intensity in CD34+ cells compared to the SM part of BMT and a homozygous variant signal in granulocytes. Both *TET2* and *RUNX1* mutations were not detectable in B- and T-cells.

**Conclusion:**

We present a heterozygous triple-mutation pattern (*KIT*, *TET2*, *RUNX1*) in mast cells (SM disease part) with additional LOH of *RUNX1* in granulocytes (CMML disease part). These identified mutations allow a more detailed insight into a multistep pathogenesis which suggests a common tumor progenitor in SM-CMML.

## Background

Systemic mastocytosis (SM) rarely occurs in combination with so called “associated clonal haematological non-mast cell lineage disease” (AHNMD) [1-5]. In most instances, the AHNMD component is of myeloid origin, including acute myeloid leukemia (AML), chronic myelomonocytic leukemia (CMML) or primary myelofibrosis (PMF) [1,6-8]. Recently, *KIT*^D816V^ mutations were identified in both disease components of SM-AHNMD with the highest frequency in SM-CMML [9], suggesting a common precursor.

## Methods and findings

**Figure 1:**
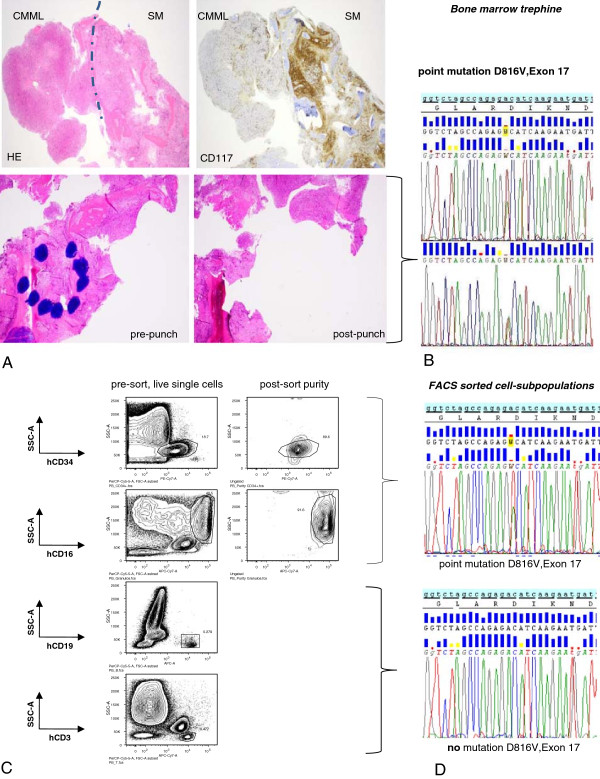
**Assessment of the *KIT***^**D816V **^**mutation on the bone marrow trephine. A***upper line*: HE stain of the bone marrow trephine showing both, CMML and SM part in distinct areas in this patient (left). A CD117 immunostain (right) highlights the mast cells in the SM compartment, however cells within the CMML compartment are negative. *Lower line*: HE control before and after punching out the SM area (containing around 90% of MC in the CD117 staining) submitted to PCR and Sanger Sequencing. **B** Forward and reverse sequences indicating the *KIT*^D816V^ mutation of microdissected SM area of the bone marrow trephine. **C** FACS sorted cells as indicated with a purity of around 90% for CD34+ stem cells and granulocytes. **D** Shows the sequencing results of the PCR amplification product for *KIT*^D816V^, and forward strands for isolated cell-subpopulations respectively.

We report on a 73 year old male patient who was initially diagnosed with CMML. Untreated for three years, extended investigations for disease progression showed a normal karyotype and neither a JAK-2 mutation nor a BCR-ABL fusion gene. Due to abnormal BM mast cell infiltrates the patient met the WHO 08 [1] criteria for SM-CMML with proven *KIT*^D816V^ mutation in different cell subpopulations as detailed in (Figure 1A-D). The patient died of disease under treatment with azacitidin (vidaza^®^) four months after this diagnosis. Autopsy findings showed additionally to the bone marrow involvement widespread disease in different extramedullary haematopoietic and visceral organs. There was no transformation into acute leukemia.

**Figure 2:**
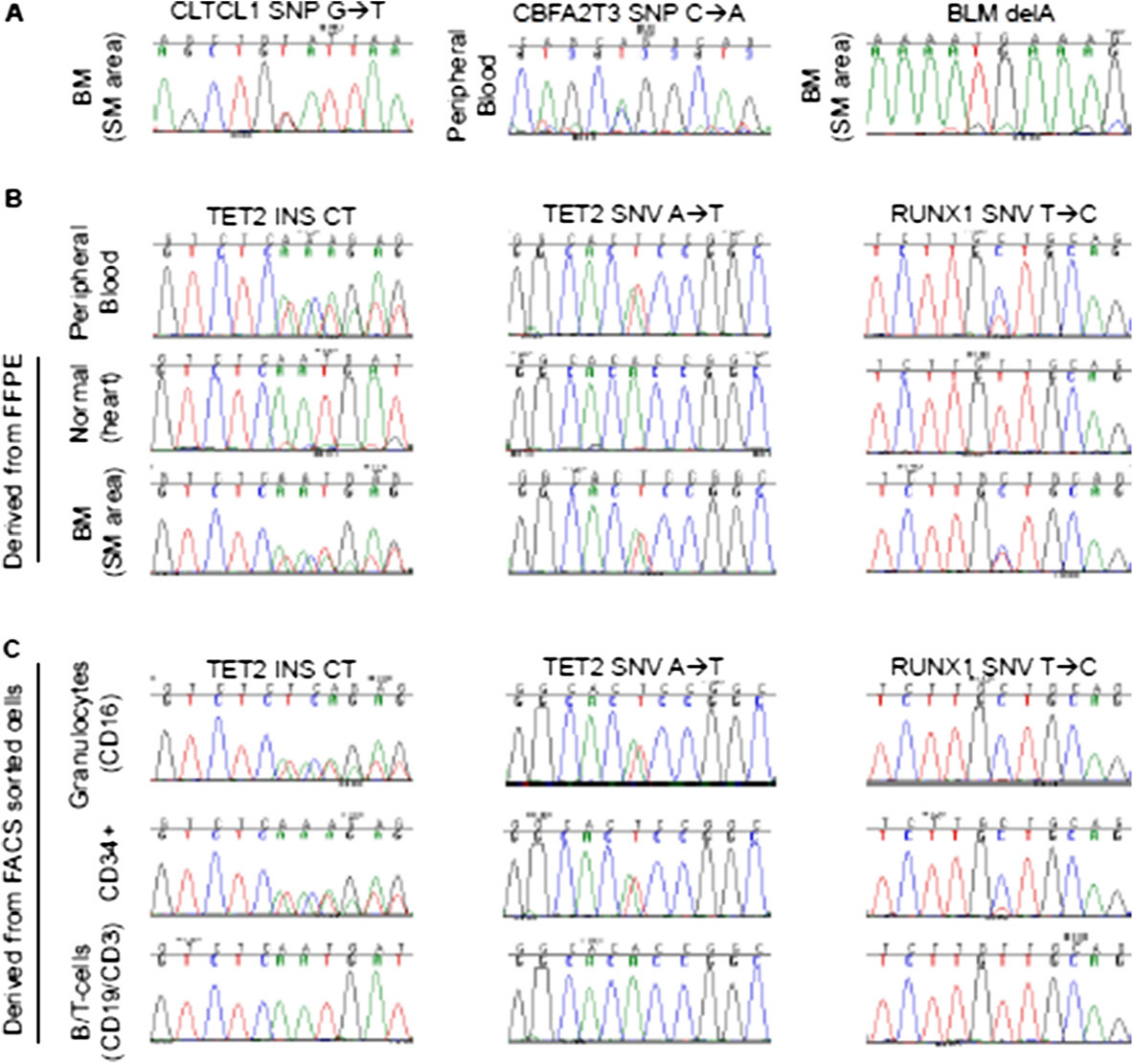
**Verification of identified variants by Sanger sequencing. A**: Representative sequences of *CBFA2T3*, *CLTCL1,* and *BLM* using DNA derived from the SM part of BMT, PB, and the SM part of BMT, respectively. **B**: Sequencing of *TET2* and *RUNX1* using DNA derived from PB, the SM part of BMT, and normal tissue. **C**: Sequencing of *TET2* and *RUNX1* using DNA derived from FACS sorted PB cell populations.

We applied a cancer gene panel containing 427 genes, custom-designed to include all exons of these genes on a solid capture array by NimbleGen (Roche). Captured targets (Additional file 1: Table S1) were deep-sequenced on a GS 454-Junior (Roche) revealing 1033 gene variants with a mean coverage of 10-fold. The analysis with the GS Reference Mapper software v.2.5 (Roche; default settings and hg19 as reference genome) identified, next to the successfully verified *KIT*^D816V^ mutation, sixty-four (6%) variants in the coding region of 42 genes (Additional file 2: Table S2). Of the 64 variants, 61 (95%) were single nucleotide polymorphisms. The remaining were two as yet unreported SNVs (*CBFA2T3* and *CLTCL1*) and one 1-bp deletion (*BLM;* COSM252959). All three mutations were confirmed by Sanger sequencing (Genetic Analyzer 3130xl (Applied Biosystems); [10]) using DNA from PB, the SM part of BMT and normal tissue, meeting the criteria for germ-line mutations (representative sequences in Figure 2A). Since, however, the assumed 1-bp deletion of *BLM* at AA515del1 – Asn515fs*16 could be identified in a non-tumoral human embryonic kidney cell line (HEK293T), we interpreted this aberration as a PCR/sequencing artefact associated with a mononucleotide repeat at this site. This finding was additionally confirmed by sub-cloning of the PCR-product in a bacterial expression vector and sequencing with different primer sets (Additional file 3: Figure S1). Interestingly, previous reports describe exactly the same deletion in gastrointestinal cancer and in T-ALL cell lines [11,12].

To achieve a higher resolution we performed a high coverage targeted re-sequencing with identical blood derived DNA using the Ion AmpliSeq™ Comprehensive Cancer Panel comprising all exons of 409 known cancer genes (Life Technologies). Forty ng DNA was used as input and the target regions were 13-fold PCR amplified. After ligation of Torrent specific adapters, the amplicons were subjected to clonal expansion on spheres using the Ion One Touch 2 system (Life Technologies). Subsequently, the amplicons were dispersed on a PI chip and sequenced on the Ion Proton platform [13]. Mean coverage was 1674-fold whereas more than 93% of the amplicons were covered at least 100-fold and more than 81% at least 500-fold allowing sensitive variant detection. Alignment (hg19), local re-alignment (2-fold), and probabilistic variant detection (90% probability and 10-fold coverage) were performed using CLC Genomics Workbench version 5.5. Additional filtering resulted in variants with following characteristics: i) non-synonymous variants and INDELs resulting in frame-shifts, ii) phred-score > = 20 and variant coverage > = 50x, and iii) forward/reverse read ratio > = 0.25. These filter criteria resulted in identifying 177 variants (Additional file 4: Table S3). From these, 144 were present in the 1000 Genome database and dbSNP and were therefore excluded in the following analysis. Under additional more stringent filtering criteria for 1-bp INDELs in homopolymeric regions (CLC default settings for 454/Ion) a final list of 11 variants emerged (Additional file 5: Table S4) including *KIT*^D816V^. Interestingly, variants of two genes previously described in SM-AHNMD [14]*TET2* (p.Thr1027fs, p.Cys1263Ser) and *RUNX1* (p.Asn109Ser) were detected. Although detectable by the 454-Junior, their coverage was too low to be called by the GS Reference Mapper. Of note, 44 variants of the 64 variants identified by the 454-Junior/GS Reference Mapper platform lay in target regions of the CCP (Additional file 2: Table S2). From these, 35 were verified by the Ion Torrent/CLC platform, one was differently aligned, and eight were not detected.

Thus, the variants in *TET2* and *RUNX1* represented most probably true positive calls potentially involved in SM-CMML pathogenesis. Although, they have not yet been reported in the Cosmic database, the mutations highly likely influence the protein function: i) the frame-shift mutation in *TET2* occurring in front of the two conserved regions of TET2 (1104–1478, 1845–2002) and the SNV lying within the first region [15], ii) the mutation in RUNX1 located in the RUNT domain which is responsible for proper DNA binding and heterodimerization [16].

The 2-bp insertion (p.Thr1027fs) and the SNV (p.Cys1263Ser) in *TET2*, and the SNV (p.Asn109Ser) in *RUNX1* were confirmed in PB and the SM part of BMT (Figure 2B). Very low signals of the variants were detected in normal tissue, most probably due to infiltrating mast cells. Whereas no signals were found in B- and T-lymphocytes, similar signal intensities for both *TET2* mutations were seen in CD34+ precursor cells and CD16+ granulocytes comparable to the signals in the SM part of BMT (Figure 2C). In contrast, *RUNX1* exhibited a double intensity of the variant in CD34+ cells compared to the SM part of BMT and a homozygous variant signal in granulocytes.

**Figure 3:**
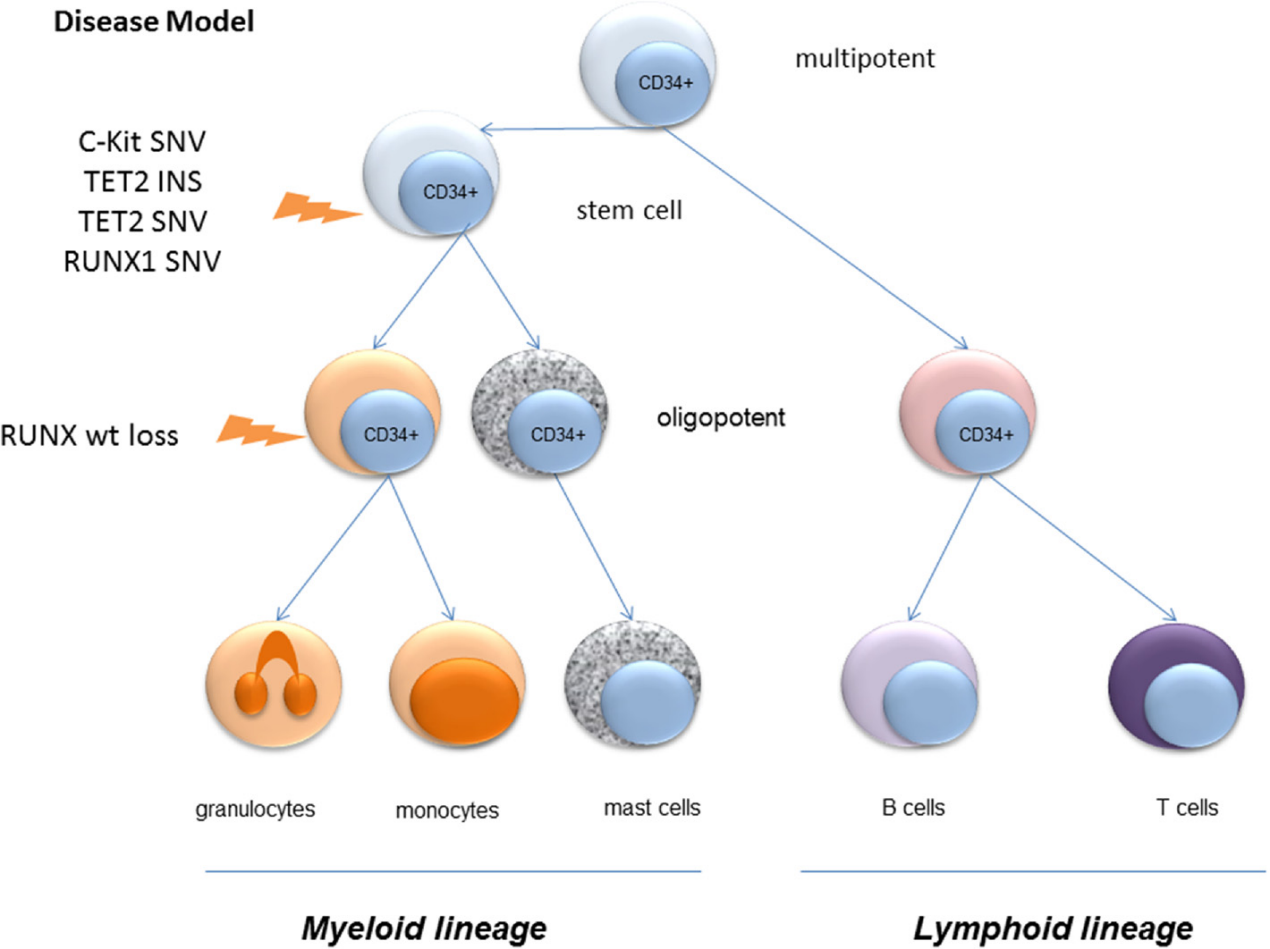
Hypothetical disease model of SM-CMML pathogenesis.

Inasmuch the SNPs and seven variants identified (Additional file 5: Table S4) are implicated in the disease development needs to be confirmed. Importantly however, we present an in-depth analysis of the sub-clonal relation of SM-CMML which reveals essential concomitant key mutations in *KIT*, *TET2*, and *RUNX1* in the emergence of different disease compartments. Since the mutant allelic fraction in the blood of *KIT* (0.44), *TET2* (0.45, 0.44), and *RUNX1* (0.53) assessed by NGS (Proton platform) were in a similar range (Additional file 5: Table S4), we assume that these mutations belong to the same CD34+ sub-clonal cell population. This is supported by the fact that the electropherograms of the Sanger sequencing exhibited the same intensities for *KIT* and *TET2* mutations in blood and the SM-area as well as in the sorted CD34+ and granulocytic cell fraction (Figure 2). Additionally, *RUNX1* mutant signals assessed by Sanger sequencing were highest in the granulocytic cell fraction and balanced with wild-type in the SM-area (purity 90%) which implies that an additional LOH of *RUNX1* occured in a CD34+ mast cells precursor. This is as well reflected by the fact that the mutant allelic fraction of *RUNX1* (0.53) in the blood assessed by NGS (Proton platform) was increased by 0.09 in comparison to the other three SNVs (mean mutant allelic fraction *KIT*, 2x *TET2*: 0.44). Since the KIT (CD117) protein is expressed in the SM, but not in the CMML compartment (Figure 1) ascribes the *KIT*^D816V^ mutation a pivotal role in the disease initiation, particularly of the SM fraction. Whereas *TET2* and *RUNX1* were necessary sequential mutations for progression to overt SM, homozygous loss of wild-type *RUNX1* seemingly was the driver mutation enabling a clonal expansion of the CMML lineage as suggested by our *disease model* (Figure 3). This further underlines the concept of common tumour progenitor cells leading to a spectrum of the same disease rather than two different ones as the term SM-AHNMD evokes. The presence of *RUNX1* and *TET2* mutations in mature granulocytes and in MC of the BMT in our study is quite novel, since so far only one recent study showed concomitant mutations of *KIT* and *TET2* in mast cells and granulocytes in SM-AHNMD [14]. The increased number of driving-mutations could explain the rapid clinical progression in our patient and is in line with the previous study, advocating additional molecular aberrations a prognostic role [14].

In summary, the current SM-CMML derived from the same mutated tumour progenitor cells (*KIT* SNV, *TET2* frame-shift, *TET2* SNV, *RUNX1* SNV) which acquired additional mutations (i.e. *RUNX1* wild-type loss) in different sub-clones as hypothesised in our model to finally manifest in the pathological state of SM-CMML.

## Consent

The study was approved by the official authorities of the ethical committee of the County of Zurich (StV2-2007) and a written consent was obtained by the patient.

## Abbreviations

AHNMD: Associated clonal hematologic non-mast cell lineage disease; BMT: Bone marrow trephine; CMML: Chronic Myelomonocytic Leukemia; PB: Peripheral blood; SM: Systemic Mastocytosis; SNP: Single nucleotide polymorphism; SNV: Single nucleotide variation.

## Competing interests

The authors declare that they have no competing interest.

## Authors’ contribution

MR is responsible for the NGS and the related verification analysis and wrote the manuscript. AB performed NGS and Sanger for NGS verification. QZ performed data analysis. RM performed FACS analysis. TR and DRZ did the microdissection and *C-KIT* analysis of all samples and contributed to the manuscript. JSG did the molecular BCR-ABL analysis and wrote the manuscript. MT did the initial diagnosis of SM-CMMLs, designed the study and wrote the manuscript. MGM, HM and PJW wrote and contributed to the manuscript. All authors read and approved the final manuscript.

## Supplementary Material

Additional file 1: Table S1 Target regions of the NimbleGen capture array and the AmpliSeq Comprehensive Cancer panel. Click here for file

Additional file 2: Table S2 Variants in the coding region identified by 454 GS Junior and overlap with the variants identified by the AmpliSeq Comprehensive Cancer panel and Ion Proton sequencing. Click here for file

Additional file 3: Figure S1 *BLM* Sanger sequencing. The index patient, the cell line HEK293T, and the sub-cloned PCR product of *BLM* were Sanger sequenced with *BLM* primers. The same sub-cloned *BLM* PCR product was as well sequenced with M13 primers located in the cloning vector. Click here for file

Additional file 4: Table S3 Variants detected by the AmpliSeq Comprehensive Cancer panel and Ion Proton sequencing. Click here for file

Additional file 5: Table S4 Filtered somatic variants potentially involved in SM-CMML pathogenesis detected by the AmpliSeq Comprehensive Cancer panel and Ion Proton sequencing. Click here for file
