# Supplementary material for: Modelling of a genetically diverse evolution of Systemic Mastocytosis with Chronic Myelomonocytic Leukemia (SM-CMML) by Next Generation Sequencing
Source: Exp Hematol Oncol. 2014 Jul 11;3:18. doi: 10.1186/2162-3619-3-18 (PMC4100747; doi:10.1186/2162-3619-3-18)
Supplement: Additional file 3: Figure S1 — BLM Sanger sequencing. The index patient, the cell line HEK293T, and the sub-cloned PCR product of BLM were Sanger sequenced with BLM primers. The same sub-cloned BLM PCR product was as well sequenced with M13 primers located in the cloning vector. [file 2162-3619-3-18-S3.pptx]

## Slide 1
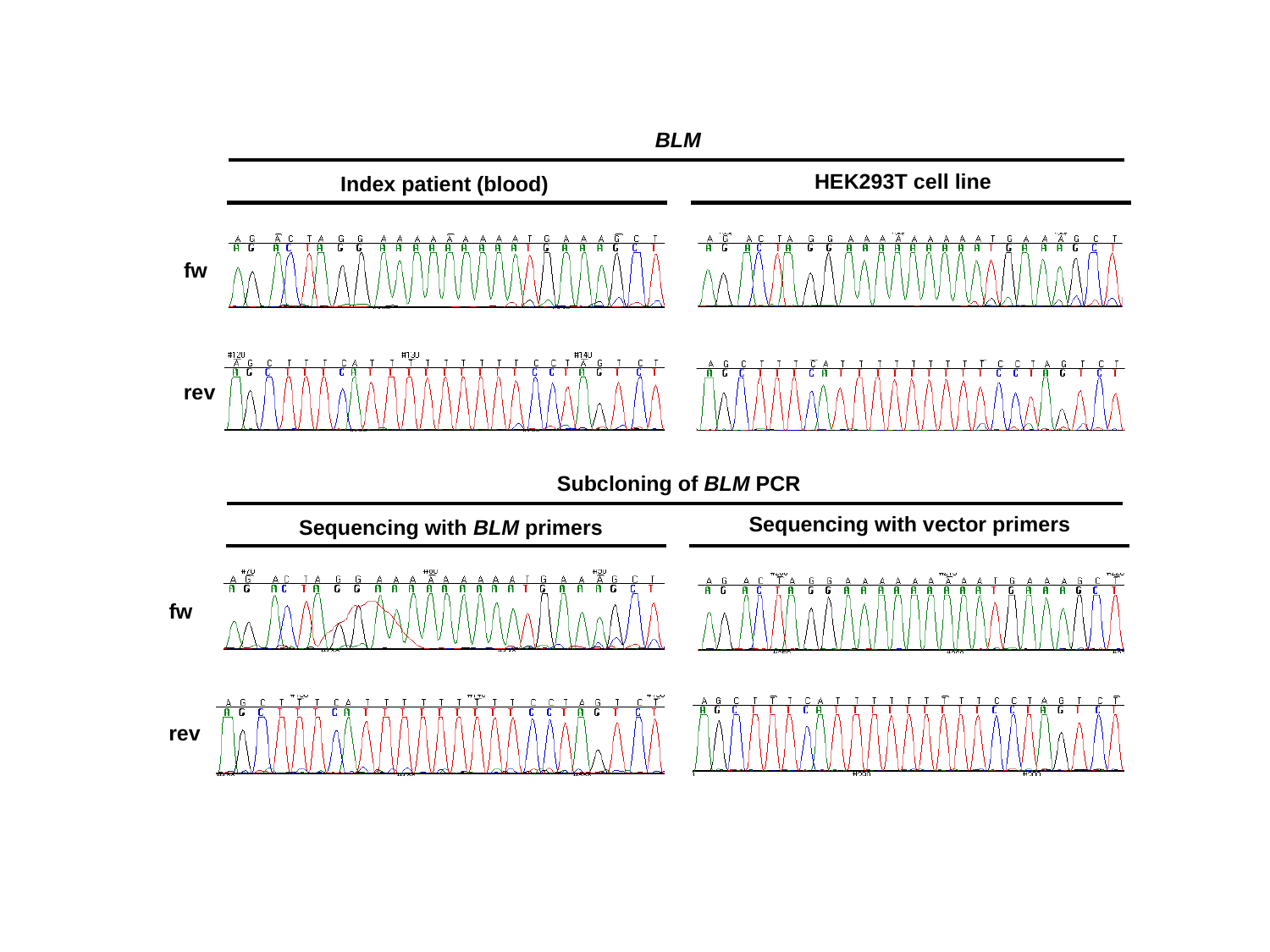

BLM
HEK293T cell line
Index patient (blood)
fw
rev
Subcloning of BLM PCR
Sequencing with vector primers
Sequencing with BLM primers
fw
rev
